# From Clinical Benefit to Economic Value: A Scoping Review of Machine Perfusion in Solid-organ Transplantation

**DOI:** 10.1097/TP.0000000000005782

**Published:** 2026-06-25

**Authors:** Pauline Breuer, Esther de Bekker-Grob, Jan IJzermans, Lucas Goossens

**Affiliations:** 1 Department of Health Technology Assessment, Erasmus School of Health Policy & Management (ESHPM), Erasmus University Rotterdam, Rotterdam, The Netherlands.; 2 Department of Surgery, Erasmus MC Transplant Institute, Rotterdam, The Netherlands.

## Abstract

Despite growing awareness of organ donation, the persistent shortage of transplantable organs remains a challenge. Machine perfusion (MP) is a preservation technique that increases organ availability. As these technologies evolve, robust economic evaluations offer an opportunity to guide timely and consistent decision-making in transplantation. Therefore, the aim was to investigate existing economic evaluations of MP to determine to what extent they capture all relevant outcomes and mechanisms. Using JBI and PRISMA guidelines, a 3-step approach was used to identify (1) all MP-related outcomes reported in studies, (2) evaluate how these outcomes were incorporated into economic evaluations, and (3) examine the mechanisms used in economic models. Of 902 references, 43 studies were included. We found that (1) the beneficial outcomes of MP extended beyond complications, including reduced waitlist mortality, lower posttransplant costs, and improved logistics; (2) the incorporation of these outcomes varied within and among organ types, no study applied a societal perspective, and models differed in cycle length, time horizon, and health states; and (3) waitlist and complication effects were inconsistently modeled. More exhaustive and transparent use of mechanisms in economic evaluations in transplant care is needed to reach optimal decisions in policy, clinical practice, and research investments.

## Graphical abstract

**Figure:**
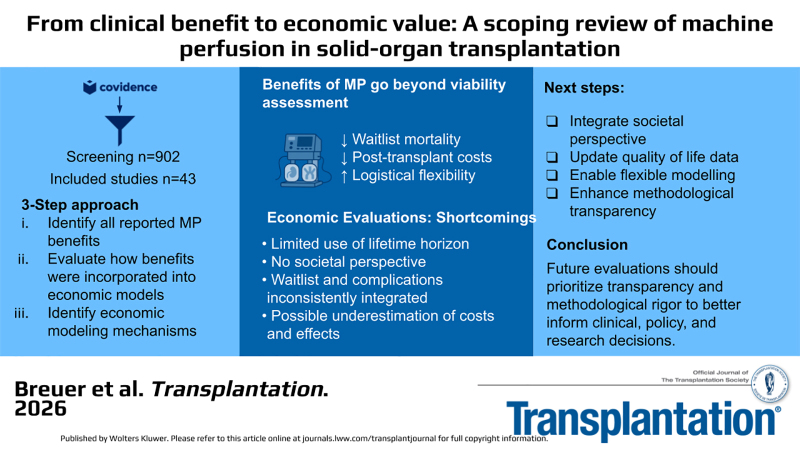

## INTRODUCTION

Although societal awareness surrounding organ donation is growing, there continues to be a shortage of organs available for transplantation.^1^ Alternative treatments are often temporary with detrimental effects on patients’ quality of life, whereas transplantation is typically the most cost-effective treatment option, especially for kidney and end-stage heart failure.^2,3^

Organ transplantation is a time-sensitive matter; once an organ is recovered, every minute counts.^4^ For the past decades, static cold storage (SCS) has been the golden standard for preservation, which comes at a low cost but cannot regenerate nor help assess the organ before transplantation.^5^ To address the limitations of SCS, machine perfusion (MP) is increasingly used to extend assessment time.^6^ Normothermic machine perfusion allows for ex situ organ assessment at physiological conditions, while hypothermic MP is performed at 4–10 °C.^5,6^ Because of to these testing and extended preservation abilities, MP has the potential to expand the donor pool through the use of extended criteria donors and organ repair, hence facilitating improved donor–recipient matching.^7^ Such advances have driven the growing adoption of MP across multiple organ types, particularly in liver and kidney transplantation.^8,9^

As the body of clinical evidence on MP grows, there is an opportunity to ensure that economic evaluations develop in parallel. Economic evaluations assess the value of new medical interventions for patients and society by comparing their costs and health outcomes to existing standards of care.^10^ In the context of MP, economic evaluations help determine whether improvements in organ preservation and transplant outcomes justify additional costs, thereby supporting transparent and evidence-based decisions about adoption and funding. These analyses often require additional metrics, as not all biologically relevant measures directly reflect patient well-being. Furthermore, complex health-economic modeling can be required to combine information from various sources and to extrapolate short-term outcomes to long-term outcomes, such as with quality-adjusted life years (QALYs).^10,11^ For transplantation, economic evaluations can be particularly complex because to organ availability dynamics, organ-specific pathways, the combinations of mechanisms through which treatments may affect outcomes, ethical considerations, and the involvement of multiple stakeholders across donation, preservation, and transplantation processes. These challenges are exacerbated in health systems, where transplant financing and reimbursement mechanisms are not fully aligned with the broader healthcare system. The United States illustrates 1 such setting with particularly fragmented transplant financing, though similar complexities exist across jurisdictions. Economic evaluations are most reliable and useful when they contain all relevant mechanisms and are performed appropriately.

Hence, the aim of this scoping review was to investigate existing economic evaluations of MP and to determine to what extent they capture all relevant outcomes and mechanisms. First, we systematically identified the range of outcomes reported in clinical and economic studies of MP across solid organ transplantation. This ensured that our review captured clinically relevant outcomes that could influence economic evaluations, including those that may or may not be incorporated into existing models. Second, we examined how identified outcomes were represented in economic evaluations. Finally, we extracted the mechanisms through which the identified outcomes were captured in economic models.

## MATERIALS AND METHODS

We followed JBI methodology and the PRISMA-ScR checklist to systematically explore comparators, methodologies, outcomes, and clinical data utilized in solid organ transplantation and MP studies.^12,13^

Adherence to JBI guidelines ensured structured data extraction and synthesis, allowing comprehensive literature review and identification of key trends. Title, abstract, and full-text screening were independently conducted by PB and LG, followed by data extraction. Disagreements were resolved without a third reviewer. Covidence^14^ software was used to facilitate screening, and data extraction followed a predefined list, with additional items added as needed.

### Search Strategy

The search strategy systematically identified literature on clinical studies and economic evaluations of MP in solid organ transplants. References of included studies were also cross checked. Searches in Medline, Embase, and Web of Science were processed by the Erasmus Medical Centre Library; duplicates were removed; and results were imported into Covidence. Complete search strategies for each database are provided in the Supplemental Digital Content (https://links.lww.com/AA/F816).

### Inclusion and Exclusion Criteria

The search included publications between January 2015 and July 2025, capturing the period surrounding the landmark 2016 study that reported the first human transplant using MP.^15^ This included economic studies conducted before MP became widely available to obtain a more comprehensive picture of potential costs. Eligible studies examined MP-related outcomes beyond the transplant procedure, including patient-related outcomes. Mid- to long-term outcomes were defined as ≥7 d posttransplant; studies with shorter follow-up were excluded. Only English-language studies on kidney, heart, liver, lung, or pancreas transplants in humans were included (**Table S1, SDC,** https://links.lww.com/AA/F816).

Interventions included various MP devices and SCS, which represent the main preservation strategies evaluated for effects on graft and patient survival, complications, and costs. Outcomes included QALYs, incremental cost-effectiveness ratio (ICER), and clinical results. Eligible clinical studies encompassed randomized and nonrandomized (prospective or retrospective) designs, while economic studies included both modeling and nonmodeling approaches.

Clinical studies were included because they provide direct evidence of MP’s benefits, such as graft survival and complications, which can directly impact cost-effectiveness. Incorporating these studies ensures the review captures both the clinical evidence and its relevance for economic evaluations, offering a comprehensive synthesis. Literature reviews were excluded.

### Data Analysis and Extraction

A 3-step approach was used:

(1) We systematically identified and listed the clinical and economic outcomes that were mentioned in the literature.
(2) We identified the key methodological characteristics of the economic evaluations (ie, study perspective, terminology, method and structure, health-related quality of life [HRQoL], health states, time horizon, and cycle length) of both model-based (defined as decision analytic models that provide a structure, such as a decision tree or Markov model, to simulate patient pathways) and nonmodel-based studies. The Consolidated Health Economic Evaluation Reporting Standards (CHEERS)^16^ guideline was used to define characteristics of these models, providing standardized recommendations to ensure transparency, consistency, and comparability across studies. Costs were categorized into 4 perspectives^16,17^: societal, healthcare, institutional, and payer. The societal perspective is the broadest, including all relevant costs regardless of who incurs them, such as patient expenses (eg, out-of-pocket payments), productivity losses (eg, absence from work or reduced productivity), and healthcare resource use.^17,18^ Healthcare costs are a subset of societal costs, capturing the societal value of resources used within the healthcare sector, including staff time, facilities, and consumables but excluding costs borne directly by patients or society outside the sector.^17^ Based on reported cost components in the included studies, we defined the institutional perspective as being restricted to resources consumed within the hospital or transplant center, such as personnel, consumables, and overhead, reflecting the value of resources required to deliver care. Finally, in contrast to the 3 aforementioned perspectives, the payer perspective focuses on costs reimbursed by insurers or other third-party payers and does not necessarily reflect actual resource use.^17^
(3) We extracted the mechanisms through which MP’s benefits were captured in economic models in addition to potential mechanisms identified in step 1. Mechanisms were defined as model features representing clinical or system processes that could alter the relative performance of strategies and were considered present if parameters influenced downstream outcomes or costs, regardless of differing values among strategies.

## RESULTS

**FIGURE 1.**
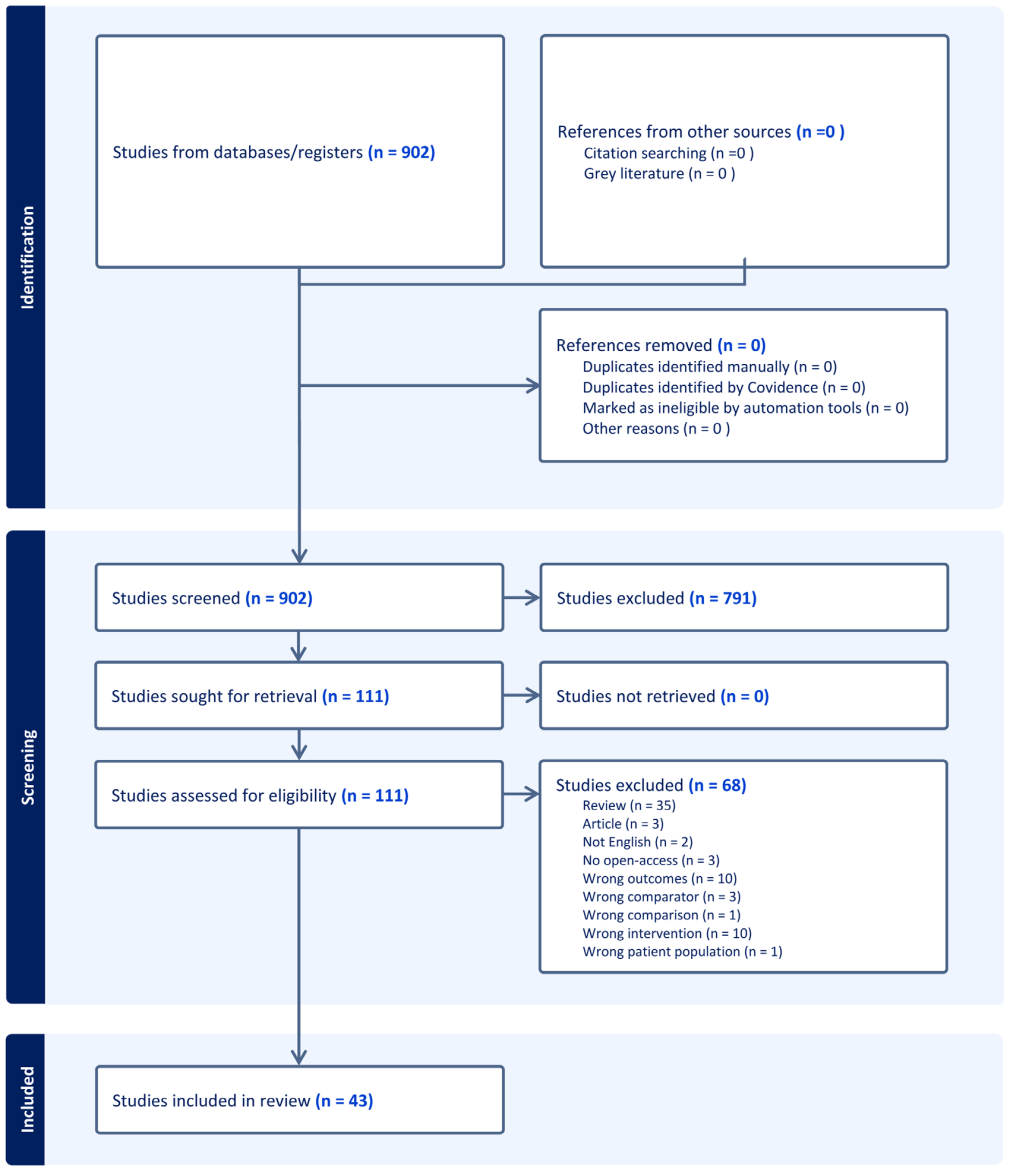
PRISMA chart. Flow diagram illustrating the study selection process. The diagram details several records identified, screened, excluded, and included at each stage according to PRISMA 2020 guidelines. Duplicates were removed before importing to Covidence.

The search resulted in n = 902 studies, of which n = 43 were included for data extraction (see Figure 1).

### Study Characteristics

Across the included studies, 16 were categorized as clinical studies (S1–16), 25 as economic studies (S17–41), of which 11 included a model (S17, S19–20, S24, S26–28, S33, S36–37 S39), one study evaluated transplant capacity (S42), and another logistical changes (S43) (see Table 1). MP was most compared with SCS (n = 28), as it is the standard of care in many transplant centers. However, more recent studies have started to compare different MP strategies directly, rather than solely contrasting MP with SCS.

**TABLE 1.** Study characteristics

Organ type	Author and year^a^	Type of study	Country	Intervention	Comparator
Kidney	S1: O’Callaghan et al (2016)	Clinical	UK	Marshall’s solution	Wisconsin’s solution
	S17: Tedesco Silva et al (2018)	Economic	Brazil	Hypothermic	SCS
	S18: Axelrod et al (2019)	Economic	USA	Hypothermic	NMP
	S19: Tedesco Silva et al (2024)	Economic	Brazil	MP of ECD	Standard criteria donor
Heart	S20: Ontario HTA (2020)	Economic	Canada	NMP	SCS
	S21: Urban et al (2023)	Economic	USA	DCD, NMP, and TA-NRP	DBD, SCS
Liver	S2: Taylor et al (2018)	Clinical	UK	DCD	DBD
	S3: Liu et al (2021)	Clinical	USA	Two pump perfusion NMP	One pump perfusion NMP
	S4: Hann et al (2022)	Clinical	UK	Suboptimal graft via NMP	SCS (group 1 and group 2)
	S5: Abbas et al(2024)	Clinical	UK	De-fattening NMP	NMP
	S6: De Vries et al (2019)	Clinical, Protocol RCT	Netherlands	DHOPE-COR_NMP	SCS
	S7: Nasralla et al (2018)	Clinical, RCT	UK	NMP	SCS
	S8: Czigany et al (2021)	Clinical, RCT	Germany, Czech Republic	Hypothermic oxygenated MP	SCS
	S9: Markmann et al (2022)	Clinical, RCT	USA	OSC (hypothermic)	ICS
	S10: Chapman et al (2023)	Clinical, RCT	USA	Hypothermic	SCS
	S22: Raigani et al (2020)	Economic	USA	Discarded livers, perfusion	NMP
	S23: Rayar et al (2020)	Economic	France	HOPE for ECD (DBD only)	SCS with ECD
	S24: Javanbakht et al (2021)	Economic	UK	NMP	SCS
	S25: Webb et al (2021)	Economic	Canada	NMP	–
	S26: Webb et al (2022)	Economic	Canada	NMP	SCS
	S27: Zimmermann et al (2022)	Economic	UK	NMP	Hypothermic
	S28: Handley et al (2023)	Economic	USA	SCS and NMP of discarded organs	SCS
	S29: Endo et al (2024)	Economic	Netherlands	DHOPE	SCS
	S30: (a) Wehle et al (2024)	Economic	USA	NMP waitlist	Pre-NMP waitlist
	S31: (b) Wehrle et al (2024)	Economic	USA	NMP of Open offers	Pre-NMP
	S32: (c) Wehrle et al (2024)	Economic	USA	NMP	SCS
	S33: Wehrle et al (2025)	Economic	USA	Flavin mononucleotide marker for NMP	Standard marker NMP and SCS
	S34: Pradat et al (2023)	Economic, Protocol RCT	France	HOPEx of ECD	SCS
	S35: Risbey et al (2025)	Economic and Pilot study	Australia	Low cost HOPE (COARO)	Standard HOPE
	S42: Toro-Díaz et al (2015)	Other	USA	Transplant capacity DCD	_
	S43: Li et al (2024)	Other	Switzerland	NMP	_
Lung	S11: Fildes et al (2015)	Clinical	UK	EVLP in marginal	Standard without EVLP
	S12: Zeriouha et al (2016)	Clinical	UK	OCS	SCS
	S13: Van Raemdonck et al (2019)	Clinical	Belgium and international database	DCD NMP	DBD
	S14: Nilsson et al (2019)	Clinical	Sweden and Denmark	EVLP	SCS
	S15: Peel et al (2023)	Clinical	Canada	EVLP	SCS
	S36: Fisher et al (2016)	Economic	UK	EVLP	SCS
	S37: McMeekin et al (2019)	Economic	UK	EVLP	SCS
	S38: Halpern et al (2021)	Economic	USA	EVLP	SCS
	S39: Peel et al (2023)	Economic	Canada	EVLP	SCS (pre-EVLP era)
	S40: Kent et al (2024)	Economic	USA	EVLP	SCS
	S41: Yin et al (2025)	Economic	USA	EVLP	Non-EVLP (Standard)
Pancreas	S16: Richards et al (2021)	Clinical	UK	NRP	sDCD

Table 1 summarizes the key characteristics of included studies, including organ type (eg, kidney, heart, liver, lung, or pancreas), first author and year of publication, country in which the study was conducted, intervention (the machine perfusion strategy evaluated), and comparator (the alternative preservation strategy against which machine perfusion was assessed, typically SCS or another perfusion approach). Studies are referenced in the text using S1–S43, where S1–S16 represent clinical studies, S17–S41 represent economic studies, and S42–S43 represent other study types.

a The references mentioned above are available in Supplemental Digital Content (https://links.lww.com/AA/F816).

DBD, donation after brain death; DCD, donation after circulatory death; DHOPE, dual hypothermic oxygenated perfusion; ECD, extended criteria donor; EVLP, ex vivo lung perfusion; HMP/MP, hypothermic machine perfusion; HOPE, hypothermic oxygenated perfusion; ICS, in situ cold storage; NMP, normothermic machine perfusion; NRP, normothermic regional perfusion; OCS, organ care system; SCS, static cold storage; sDCD, standard donation after circulatory death; TA-NRP, thoracoabdominal normothermic regional perfusion.

Most studies were conducted in high-income countries, highlighting the financial and infrastructural demands associated with implementing MP in clinical practice. Most were based in Europe (the United Kingdom, France, the Netherlands, Germany, the Czech Republic, Switzerland, and Belgium), North America (Canada and the United States), and 2 from Brazil. Most studies focused on liver transplantation, while evidence for MP in kidney, heart, lung, and particularly pancreas (S16) remains limited or emerging.

### Step 1: Benefits of Machine Perfusion

This section summarizes and compares the outcomes reported in clinical studies and economic evaluations. Clinical studies most frequently measured 12-mo graft survival, followed by complications such as early allograft dysfunction (Table 2). Economic evaluations more frequently reported resource use outcomes, such as hospital length of stay. Two clinical studies and 3 economic evaluations reported delayed graft function (**Table S6, SDC,** https://links.lww.com/AA/F816).

**TABLE 2.** Proportion of studies reporting outcomes measures

Outcomes	Study type		
	Clinical (n = 16)	Economic (n = 25)	Other (n = 2)
Length of stay ICU	8 (50%)	13 (52%)	0 (0%)
Length of stay hospital	6 (38%)	10 (40%)	0 (0%)
Primary nonfunction	6 (38%)	8 (32%)	0 (0%)
Early allograft dysfunction	7 (44%)	5 (20%)	0 (0%)
Delayed graft function	2 (13%)	3 (12%)	0 (0%)
Graft rejection	4 (25%)	4 (16%)	0 (0%)
Graft loss	4 (25%)	7 (28%)	0 (0%)
12-mo graft survival	7 (44%)	3 (12%)	0 (0%)
Retransplant	4 (25%)	3 (12%)	0 (0%)
Organ discard and utilization	5 (31%)	5 (20%)	1 (50%)

Outcomes measured include indicators of short- and long-term graft function and patient recovery. “Clinical” studies primarily report clinical outcomes, while “economic” studies perform economic evaluations. Percentages reflect the proportion of studies within each study type reporting the specified outcome. Ordered by immediate perioperative resource use, immediate graft outcomes, intermediate graft outcomes, and system-level outcomes. One study in the “other” category focused on logistical considerations (eg, timing of transplantation); therefore, the specific clinical outcomes included in this review were not reported.

For certain short-term outcomes DGF, parameter inputs in economic evaluations were not always directly reported in the corresponding clinical studies. In some cases, estimates were derived from additional sources, including earlier randomized controlled trials, single-center datasets, registry data (eg, UNOS), or supplementary literature. This reflects common practice in economic modeling, where parameters may be informed by a combination of clinical and external data sources.

DGF, delayed graft function; ICU, intensive care unit.

**FIGURE 2.**
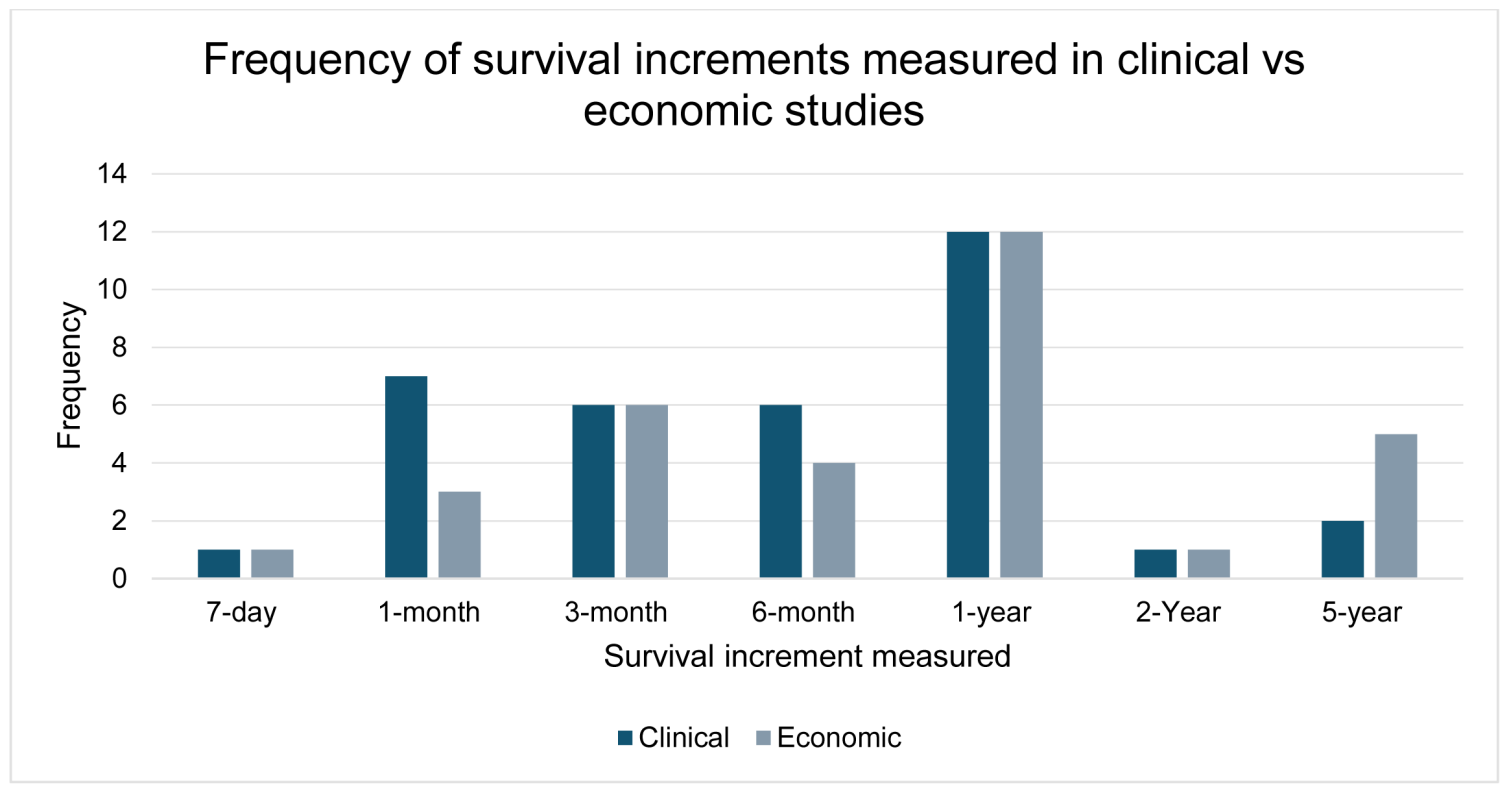
Frequency of survival increments measured in clinical vs economic studies. Survival increments refer to the time points at which survival was reported. “Clinical” indicates studies primarily reporting clinical outcomes, and “Economic” refers to studies conducting an economic evaluation. Percentages represent the proportion of studies within each type reporting the specified survival increment. One-year survival was equally reported in clinical and economic studies (50% each), while 5-y survival was more frequently reported in economic evaluations (71%) compared with clinical studies (29%), highlighting the emphasis on longer-term outcomes in cost-effectiveness analyses. Studies categorized as “Other” did not include survival, as it modeled organ supply, waitlist size or logistic and were therefore excluded in this figure.

Economic evaluations more frequently reported longer-term survival, particularly 5-y outcomes (71% of economic studies versus 29% of clinical studies), highlighting the importance of sustained beneficial outcomes for cost-effectiveness modeling (see Figure 2). Reporting of 3-mo survival was balanced across study types (50% each), while 1-y survival was equally reported, underscoring its relevance for the period with the highest risk of complications (**Table S6, SDC**, https://links.lww.com/AA/F816).

Clinical studies emphasized biological and functional outcomes such as early allograft dysfunction. Notably, 12-mo graft survival was included in 44% of clinical and 12% of economic studies. Economic studies focused on patient survival to measure cost-effectiveness. Outcomes such as PNF, retransplantation, graft rejection, and graft loss were reported with similar frequency (Table 2).

An additional outcome included in both study types were discard rates and utilization rates. Several studies (S7, S10, S14, and S22) reported these outcomes to capture how MP may influence organ use, while others examined acceptance or rejection patterns of donor organs (**Table S3, SDC**, https://links.lww.com/AA/F816).

An additional benefit reported in Li et al (S43) was shifting liver transplants to elective procedures. This approach can improve organ utilization, donor–recipient matching, and surgical planning, potentially enhancing patient outcomes and optimizing healthcare resource use.

### Step 2: Economic Evaluation Types and Methodologies

#### Study Perspective

According to CHEERS^16^ and Drummond et al,^17^ studies should report the perspective of the analysis to indicate which costs and outcomes are included. Four economic evaluations (S24, S27, and S36–37) adopted a healthcare perspective (16%), which extends beyond institutional costs but still excludes indirect costs such as productivity losses. The most used perspective was institutional (52%), focusing on expenses incurred in the first year (S21–23, S29, S30–33, and S38–41). A payer perspective was used in 32% of studies (S17–20, S25–26, S28, and S34). None of the studies used a societal perspective; hence, they did not account for productivity losses or informal care, which could impact cost-effectiveness results (**Table S2, SDC**, https://links.lww.com/AA/F816).

All studies adopting a healthcare perspective explicitly stated both the perspective and the costs considered, including perfusion costs and posttransplant care (S24, S27, and S36–37). Three studies accounted for waiting list costs (S27 and S36–37), and Fischer et al (S36), McMeekin et al (S37), and Javanbakht et al (S24) included outpatient costs, covering both clinic and GP visits (**Table S2.1, SDC**, https://links.lww.com/AA/F816). Studies using an institutional perspective rarely reported it explicitly (S23, S29, S33, and S39). For the remaining studies, the perspective was inferred according to the definition of an institutional perspective by examining the types of costs captured, which primarily included direct costs borne by the transplant center, such as perfusion devices, staffing, and immediate perioperative care (**Table S2.2, SDC**, https://links.lww.com/AA/F816). While Wehrle et al explicitly mentions an institutional perspective, they report “hospital costs of medical care,” hence a payer perspective via tariffs could not be ruled out (S33). In studies adopting a payer perspective (S17–19, S20, S25–26, S28, S34), the perspective was consistently stated and focused on reimbursable expenditures, covering hospitalizations, device costs, and postdischarge outpatient care (**Table S2.3, SDC**, https://links.lww.com/AA/F816).

While most studies evaluated the costs and effects of MP compared with SCS, Wehrle et al (S30) reported costs and health outcomes across different allocation systems, whereas Raigani et al (S22) focused specifically on the costs of identifying viable organs rather than total treatment costs.

#### Terminology, Method, and Structure

CHEERS^16^ recommends that economic evaluation results be reported using standardized terminology, with terms such as “cost-effective,” “cost-saving,” and “ICER” applied consistently and according to their conventional definitions. In the study by Endo et al (S29), cost-effectiveness methods and terminology were used unconventionally, for instance by defining cost-effectiveness as cost-saving. Kent et al (S40), rather than calculating the standard ICER, they reported a ratio of ratios, resulting in an inaccurate conceptual approach and calculations.

When modeling approaches were applied, 1 study (S17) used a decision tree (5%), 4 (S19, S27, S36–37) a Markov model (20%), and 3 studies (S24, S26, S28) combined both (15%). The studies by Fisher et al (S36) and McMeekin et al (S37) were based on the same trial and Markov model (**Table S4, SDC**, https://links.lww.com/AA/F816).

#### Use of HRQoL

CHEERS^16^ recommends that model-based economic evaluations should include HRQoL measures. Among studies reporting HRQoL, MP interventions were generally associated with higher QALYs, with the exceptions of Fischer et al (S36) and McMeekin et al (S37). This improvement in QALYs was primarily attributed to lower waitlist mortality. Most studies relied on HRQoL values from Ratcliffe et al, which were based on data collected between 1996 and 1998 (**Table S5, SDC**, https://links.lww.com/AA/F816). Kent et al (S40) used Karnofsky Performance Status at 1-y posttransplant to approximate HRQoL, assuming it remained constant over time.

#### Health States

Studies should report the health states and explain how these capture clinically important events, consistent with CHEERS recommendations.^16^ Most studies used 4 base health states: waitlist, transplant, posttransplant, and death. Some models (S17 and S27) included posttransplant complications as a separate health state.

In models that started before transplantation, mortality was modeled in 2 ways. In 1 type of model, patients had a risk of dying in each cycle while waiting for a transplant (waitlist mortality). In other models, mortality was modeled indirectly, if a patient did not receive a transplant, they were assumed to have died. For example, Tedesco Silva et al (S19) explicitly modeled waitlist mortality, while Zimmermann et al (S27) accounted for mortality because to not receiving a transplant. Peel et al (S39) included a prewaitlist referral stage to reflect distinct phases of care.

Long-term posttransplant follow-up was handled differently across studies; Peel et al (S39) grouped posttransplant years 1 and 2 together, while assigning a separate state for years 3 and beyond, aligning with hospital follow-up and surveillance protocols. Other models, however, only distinguished the first posttransplant year (**Table S4, SDC**, https://links.lww.com/AA/F816).

Peel et al (S39) included perimortem states to capture the high costs of end-of-life care, such as ICU stays and multiorgan failure. This approach aimed to improve cost estimations by explicitly modeling palliative care rather than assuming an immediate transition to death.

#### Time Horizon

Studies should report the time horizon over which costs and outcomes are evaluated, providing justification that it is sufficient to capture all relevant differences among strategies.^16^ Time horizons ranged from 1 y to lifetime, influenced by the chosen perspective. Cost–utility studies adopted longer time horizons than other types of economic evaluations. Many studies adopted shorter time horizons, often limited to the first year (S17, S34, S40). Although some studies used a 5-y time horizon (S19, S21, S27, and S39), this may still be too short to fully capture long-term clinical and economic outcomes. A lifetime horizon, as recommended by the CHEERS guidelines, was used to capture the long-term impacts of both the intervention and the comparator, ensuring inclusion of all relevant costs and outcomes in only 4 studies (S24, S27, S36, and S37; **Table S4, SDC**, https://links.lww.com/AA/F816).

#### Cycle Length

CHEERS^16^ advises that the model’s cycle length should reflect the disease or intervention’s natural history. Differences in cycle length ranged from 10 d (S39) to 1 y (S27 and S36–37). The 1-y cycle length was often justified by the elevated risk of complications within the first-year posttransplant (S19, S24, S26, S28, and S36–37). Peel et al (S39) used a 10-d cycle length, which better captured the immediate posttransplant period. A short cycle length increases model complexity, requiring a higher number of transitions and computational resources. Some studies applied a 1-mo cycle length that enhances computational efficiency while capturing relevant short- and mid-term effects. In contrast, the 1-y cycle length used by, for example, Webb et al (S26) may miss critical early posttransplant events that influence both costs and effects (**Table S4, SDC**, https://links.lww.com/AA/F816).

### Step 3: Economic Modeling Mechanisms

Nine mechanisms in 4 domains (supply, timing, perioperative resources, and posttransplant outcomes and resources) are shown in Table 3. Each mechanism captures 1 or more pathways through which clinical events and system processes influence clinical outcomes, costs, and HRQoL. These mechanisms could affect the outcomes for each intervention similarly or differently. For example, Tedesco Silva et al (S19) parameterized additional organs and discard rates differently (dark blue) for the intervention and comparator. These differences affected waitlist dynamics, as a higher organ availability or lower discard rate indirectly reduced time on the waitlist (W in the cell). Similarly, the cost of transplantation was assigned different values (dark blue) for the intervention and comparator, reflecting real-world differences in resource use, while posttransplant survival was assumed to be the same (light blue) across strategies. White cells showed when a mechanism was not explicitly mentioned and parameterized. Including these examples illustrate how specific mechanisms can drive differences in both clinical and economic outcomes across models.

**TABLE 3.**
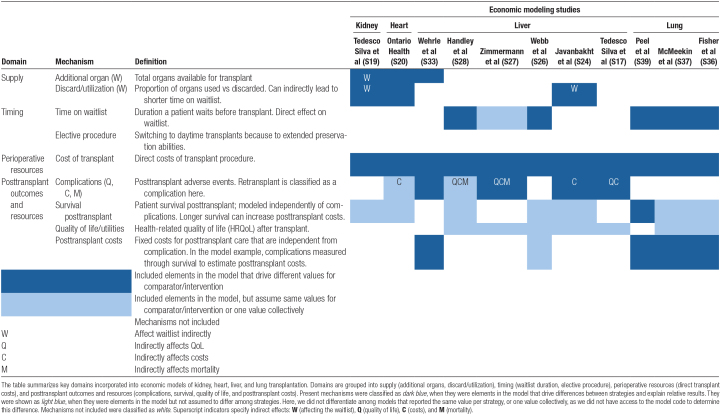
Economic modeling studies of solid organ transplantation by domain and mechanism

Ten out of 11 models considered that MP could affect waiting time (S19, S20, S33, S28, S27, S26, S24, S39, S37, and S36). Of these, 3 studies modeled additional organs (S19, S20, and S33) and 2 modeled organ discard/utilization (S19, S20, and S24). In 2 studies, the supply domain had indirect effects on the waitlist, for example, by altering the overall pool of available grafts, which in turn affects patient-level transitions from waitlist to transplantation (S19 and S24). In other studies, these supply mechanisms were not linked to the waitlist (white), indicating that changes in organ availability did not translate into different probabilities of receiving a transplant, thereby omitting waitlist dynamics (S20 and S33). In contrast, 5 models incorporated a direct representation of waitlist dynamics (dark blue), where there was a higher transition probability of moving from waitlist to transplant when MP was the intervention (S26–27, S36–37, and S39).

Zimmermann et al (S27) directly modeled waitlist, however, assumed equal time on the waitlist and equal transplant activity across strategies (light blue). Javanbakht et al (S24) took a different approach, assuming that if no organs were discarded, supply would automatically equal demand and waiting times would be eliminated. Tedesco Silva et al (S17) did not incorporate any of these domains, as their model began at posttransplantation.

None of the reviewed models included the elective procedure mechanism. As for perioperative resources, all studies included the direct cost of transplantation across kidney, heart, liver, and lung models. This consistency underscores the higher fixed costs associated with MP.

Complications were modeled in 6 out of 11 studies (S17, S20, S24, S27–28, and S33). When modeled, complications could influence estimates of HRQoL, costs, and mortality within the model (shown as Q, C, and M, respectively, in Table 3). When complications were not explicitly included, mechanisms related to survival, posttransplant outcomes, and resource use were used as proxies. For example, increased patient survival affected posttransplant costs.

Across studies, complications were the most consistently included mechanism within the posttransplant outcomes and resource use domain, though their linkage to other outcomes varied. Four studies (S17, S24, S27, and S33) modeled differences in complication rates across strategies (dark blue). Among these, Zimmermann et al (S27) explicitly modeled the downstream effects of complications on HRQoL, costs, and mortality. Handley et al (S29) and Zimmermann et al (S27) included both costs and effects of retransplant. Javanbakht et al (S24) included effects on costs, and Tedesco Silva et al (S17) included effects on HRQoL and costs. Two studies modeled complications, but applied the same values across strategies (light blue): Ontario Health et al (S20) incorporated effect on costs, and Handley et al (S28) included effects on HRQoL, costs, and mortality. Five studies did not explicitly model complications (S19, S26, S36–37, and S39); in these cases, survival or survival combined with posttransplant costs were used as a proxy to reflect complication-related effects. In studies that included survival as a modeled outcome, it was assumed to be the same across strategies (light blue), with the exception of Peel et al (S39), who incorporated different survival outcomes among strategies (dark blue).

All 7 studies (S17, S24, S26–28, and S36–37), that included HRQoL applied the same values among strategies (light blue). However, studies that linked complications to HRQoL indirectly reflected differential effects through the complications mechanism. For example, Tedesco Silva et al (S17) distinguished HRQoL values based on graft function, thereby modeling improved HRQoL within the posttransplant health state.

The 4 studies that included posttransplant costs, applied different values across strategies (dark blue; S33, S36–37, and S39), whereas Webb et al (S26) assumed identical costs across groups (light blue). Tedesco Silva et al (S19), determined posttransplant costs indirectly: fixed waitlist and transplant flows drove downstream outcomes, including costs associated with dialysis dependence, rather than assigning a separate fixed posttransplant cost. Consequently, studies that incorporated cost effects through the complications domain did not always include independent posttransplant cost parameters.

## DISCUSSION

This scoping review identified that economic evaluations of MP in solid organ transplantation often did not capture all relevant clinical benefits. However, several studies incorporated key outcomes, such as complications and, in some cases, waiting list effects. Despite limitations in transparency regarding perspective, short time horizons, lack of societal perspectives, and outdated HRQOL data, these efforts provide a starting point to explore the value of MP.

Clinical and economic studies described complementary outcomes, however, not all clinical outcomes were included in economic evaluations, and vice versa. Clinical research primarily reported short- to medium-term outcomes, while economic models often captured long-term effects, reflecting different objectives: Clinical trials focused on immediate postoperative outcomes, while economic evaluations aimed to understand how early benefits translated into sustained value for health systems. Benefits of MP included improved donor organ utilization, earlier transplantation, reductions in posttransplant complications, and logistical improvements.

While some models incorporated waitlist dynamics and pathways from organ failure to posttransplant outcomes, these features were inconsistently represented. The outcomes included typically reflected the model’s purpose; for example, budget impact analyses omitted quality-of-life.

Economic evaluations displayed substantial methodological variation, and terminology was used inconsistently, for example, describing interventions as “cost-effective” when they were cost-saving or calculating ICERs incorrectly. Additionally, the study perspective and included costs were often not explicitly reported, rendering it difficult to compare results or assess the economic impact of interventions.

Health state definitions were heterogeneous. Additionally, none of the studies adopted a societal perspective, despite its relevance in transplantation, where productivity, caregiver burden, and HRQoL can be substantial. Model cycle lengths varied, with some annual cycles that may not accurately capture the timing of posttransplant events, leading to a less accurate representation of the developments in patients’ health and treatment. For example, including productivity losses would provide a more complete picture of transplantation’s value and support better patient outcomes and resource allocation.

Notably, the short time horizons used by most studies lead to an underestimation of health and survival benefits, given that transplantation can increase life expectancy. At the same time, it also results in underestimation of some medical costs that are related or unrelated to transplantation, while potentially also missing savings from other medical costs and prevented absence from work. The balance of these is uncertain, and may differ per organ, patient group, intervention, and country.

While some studies reported key features recommended by CHEERS,^16^ such as model structure, and outcome measures, reporting was not always consistent nor transparent. For example, models that excluded important pathways, like waitlist, have underestimated the effects and at the same time overestimated MP’s costs stemming from increased patient survival. Overall, these evaluations acknowledged MP as an important innovation, but approaches had not yet fully aligned with standard recommendations for transparency and comparability in economic modeling.

The representation of the 7 mechanisms identified varied across models. Some models assumed identical HRQoL for MP and SCS or relied on outdated data, potentially underrepresenting patient benefits. Waitlist and complications were inconsistently captured: in some cases, models reflected how complications affected costs, mortality, and quality of life, while in others, identical values were applied to both the intervention and comparator, reducing sensitivity to clinically meaningful differences. Not all potential mechanisms, such as elective procedure pathways, were included. While it was not necessary for every model to incorporate all mechanisms, explicitly defining which were included could improve transparency and comparability across studies.

An earlier review by Boteon et al^19^ summarized clinical benefits and potential economic implications of MP in liver transplantation, while noting the scarcity of economic evaluations in the field. We agree with their observation regarding the limited number of studies, but our methodological review identified additional issues beyond scarcity. Many economic evaluations relied on simplified or heterogenous approaches, inconsistently captured key clinical pathways, and varied widely in their modeling of outcomes, costs, and time horizons.

Limitations include publication lag, which may have resulted in the omission of recently completed studies. Second, as models were not stratified by organ type, nuances across different transplant settings may have been underrepresented. This approach was intentional, as it allowed for a consistent comparison of modeling principles rather than clinical outcomes, but we recognize that each organ has unique clinical and logistical considerations that shape its economic representation. Finally, most models evaluated liver transplantation, which should not hinder conclusions made about the current state of evidence. The current economic evidence does not allow for robust conclusions about the cost-effectiveness of MP yet. Methodological shortcomings and inconsistent modeling approaches introduce considerable uncertainty, potentially under- or overestimating beneficial outcomes and costs.

Based on these findings, several recommendations can guide future economic evaluations of MP in transplantation. Studies should prioritize utilizing standard methods for economic evaluations, including model structures, key mechanisms, and a societal perspective. Lifetime horizons, appropriate cycle lengths, and sensitivity analyses are recommended to capture health gains, costs, and uncertainty. Hence, a flexible reference model embedding these features could facilitate evaluation of emerging therapies. Improving methodological rigor and transparency is essential to ensure that the clinical benefits of MP are accurately reflected in their economic value for decision-makers.

## CONCLUSION

Our review shows that existing economic evaluations of MP in transplantation vary in scope and often fail to capture all relevant outcomes and mechanisms, including long-term outcomes, waitlist dynamics, and societal costs. These gaps suggest that current studies may underestimate its full impact in terms of MP’s benefits. Future evaluations should prioritize transparency and methodological rigor to better inform clinical, policy, and research decisions.

## ACKNOWLEDGMENTS

The authors thank Wichor Bramer, PhD, from the Erasmus MC Medical Library for developing and updating the search strategies. We thank the editor and reviewers for their thoughtful and constructive feedback, which helped improve the clarity, rigor, and clinical relevance of our article.

## Supplementary Material


