# Supplementary material for: From Clinical Benefit to Economic Value: A Scoping Review of Machine Perfusion in Solid-organ Transplantation
Source: Transplantation. 2026 Jun 25;110(8):e1608–17. doi: 10.1097/TP.0000000000005782 (PMC13384376; doi:10.1097/TP.0000000000005782)
Supplement: Supplementary file 1 [file tpa-110-e1608-s001.pdf]

Supplemental Digital Content

Contents

Supplemental Digital Content 1
SDC, Materials and Methods, Search strategy 2
SDC Material Methods Table S1: Inclusion and exclusion criteria used for the title, abstract and full-text screening 4
SDC Results Table S2: Costs included in economic evaluations 5
SDC Results Table S2.1: Healthcare perspective costs 5
SDC Results Table S2.2: Institutional perspective costs 7
SDC Results Table S2.3: Payer perspective costs 10
SDC Results Table S3: Discard and acceptance rates of organs, reported in included studies 13
SDC Results Table S4: Characteristics: Economic studies 14
SDC Results Table S5: Availability, reference and measurement method for health-related quality of life 17
SDC Results Table S6: Clinical outcomes measured in all included studies 18
SDC: References (included studies) 21

SDC, Materials and Methods, Search strategy

Medline

(exp Organ Transplantation / OR (((organ* OR heart* OR kidney* OR liver* OR lung* OR renal* OR hepatic* OR pulmonar* OR cardiac* OR pancrea*) ADJ3 (transplant* OR retransplant* OR graft* OR donor* OR donation* OR procurement* OR recipient* OR retrieval* OR harvesting*)) OR cDCD OR cDCDD OR uDCD OR uDCDD OR DCD OR DCDs OR DCDD OR DBD OR DBDs OR NHBD OR NHBDs OR NHBOD OR NHBODs OR ECD).ab,ti,kw.) AND (exp Perfusion/ OR Extracorporeal Membrane Oxygenation/ OR Organ Preservation/ OR (((extracorporeal* OR Extrapulmonar*) ADJ3 (oxygenation* OR cardiopulmonar* OR resuscitation* OR support* OR circulation*)) OR ecmo OR ((graft* OR transplant* OR organ* OR heart* OR kidney* OR liver* OR lung* OR renal* OR hepatic* OR pulmonar* OR cardiac* OR pancrea* OR machine* OR normothermic* OR subnormothermic* OR hypothermic* OR ex-vivo* OR in-vivo* OR ex-situ* OR in-situ OR regional* OR cold* or warm*) ADJ3 (perfus* OR reperfus* OR preserv* OR storage* OR repair* OR conservation*)) OR ((intervention*) ADJ3 (donor* OR research* OR organ*)) OR NRP OR ANRP OR TANRP OR XVIVO OR OrganOx OR (control* ADJ3 oxygenat* ADJ3 rewarm*) OR NMP OR MP OR HMP OR EVMP OR ESMP OR ESHP OR SNMP OR organ-care-system* OR OCS OR D-HOPE OR COR OR OP-ECMO OR ECPR OR ECLS OR EISOR OR ISRP OR Persufflation* OR ((Cerebral* OR brain* OR systemic*) ADJ3 (circulation* OR blood-flow))).ab,ti,kw.) AND (exp Cost-Benefit Analysis/ OR Quality-Adjusted Life Years/ OR Disability-Adjusted Life Years/ OR Health Care Costs/ OR Cost of Illness/ OR Technology Assessment, Biomedical/ OR * "Costs and Cost Analysis"/ OR * "Health Care Economics and Organizations"/ OR * Economics/ OR ((economic* ADJ3 evaluat*) OR ((cost OR costs) ADJ3 (benefit* OR effectiv* OR utilit* OR compar* OR ratio OR quality-of-life OR analys*)) OR low-cost OR ((quality OR disablit* OR wellbeing OR well-being) ADJ3 adjust* ADJ3 (life-year* OR lifeyear*)) OR daly* OR qaly* OR waly* OR ((health OR biomedical*) ADJ3 technolog* ADJ3 assessment*) OR hta).ab,ti,kw. OR (economic* OR cost OR costs).ti.) NOT (exp animals/ NOT humans/) AND english.la. AND 2015:2030.(sa_year).

Embase

('organ transplantation'/exp OR 'organ donor'/exp OR 'graft recipient'/de OR (((organ* OR heart* OR kidney* OR liver* OR lung* OR renal* OR hepatic* OR pulmonar* OR cardiac* OR pancrea*) NEAR/3 (transplant* OR retransplant* OR graft* OR donor* OR donation* OR procurement* OR recipient* OR retrieval* OR harvesting*)) OR cDCD OR cDCDD OR uDCD OR uDCDD OR DCD OR DCDs OR DCDD OR DBD OR DBDs OR NHBD OR NHBDs OR NHBOD OR NHBODs OR ECD):Ab,ti,kw) AND ('perfusion and superfusion'/exp OR 'extracorporeal oxygenation'/exp OR 'graft preservation'/exp OR 'organ preservation'/exp OR 'controlled oxygenated rewarming'/de OR 'regional perfusion'/de OR 'organ perfusion'/exp OR 'graft perfusion'/de OR reperfusion/de OR perfusion/mj OR (((extracorporeal* OR Extrapulmonar*) NEAR/3 (oxygenation* OR cardiopulmonar* OR resuscitation* OR support* OR circulation*)) OR ecmo OR ((graft* OR transplant* OR organ* OR heart* OR kidney* OR liver* OR lung* OR renal* OR hepatic* OR pulmonar* OR cardiac* OR pancrea* OR machine* OR normothermic* OR subnormothermic* OR hypothermic* OR ex-vivo* OR in-vivo* OR ex-situ* OR in-situ OR regional* OR cold* or warm*) NEAR/3 (perfus* OR reperfus* OR preserv* OR storage* OR repair* OR conservation*)) OR ((intervention*) NEAR/3 (donor* OR research* OR organ*)) OR NRP OR ANRP OR TANRP OR XVIVO OR OrganOx OR (control* NEAR/3 oxygenat* NEAR/3 rewarm*) OR NMP OR MP OR HMP OR EVMP OR ESMP OR ESHP OR SNMP OR organ-care-system* OR OCS OR D-HOPE OR COR OR OP-ECMO OR ECPR OR ECLS OR EISOR OR ISRP OR Persufflation* OR ((Cerebral* OR brain* OR systemic*) NEAR/3 (circulation* OR blood-flow))):ab,ti,kw) AND ('economic evaluation'/exp OR 'quality adjusted life year'/de OR 'disability-adjusted life year'/de OR 'health care cost'/de OR 'societal cost'/de OR 'economic aspect'/de OR 'biomedical technology assessment'/de OR cost/mj OR 'economic parameters'/mj OR 'health economics'/mj OR economics/mj OR ((economic* NEAR/3 evaluat*) OR ((cost OR costs) NEAR/3 (benefit* OR effectiv* OR utilit* OR compar* OR ratio OR quality-of-life OR analys*)) OR low-cost OR ((quality OR disablit* OR wellbeing OR well-being) NEAR/3 adjust* NEAR/3 (life-year* OR lifeyear*)) OR daly* OR qaly* OR waly* OR waiting-list* OR ((health OR biomedical*) NEAR/3 technolog* NEAR/3 assessment*) OR hta):Ab,ti,kw OR (economic* OR cost OR costs):ti) NOT ([animals]/lim NOT [humans]/lim) NOT ([conference abstract]/lim) AND [english]/lim AND [2015-2024]/py

Web of science

TS=((((organ* OR heart* OR kidney* OR liver* OR lung* OR renal* OR hepatic* OR pulmonar* OR cardiac* OR pancrea*) NEAR/2 (transplant* OR retransplant* OR graft* OR donor* OR donation* OR procurement* OR recipient* OR retrieval* OR harvesting*)) OR cDCD OR cDCDD OR uDCD OR uDCDD OR DCD OR DCDs OR DCDD OR DBD OR DBDs OR NHBD OR NHBDs OR NHBOD OR NHBODs OR ECD)) AND TS=((((extracorporeal* OR Extrapulmonar*) NEAR/2 (oxygenation* OR cardiopulmonar* OR resuscitation* OR support* OR circulation*)) OR ecmo OR ((graft* OR transplant* OR organ* OR heart* OR kidney* OR liver* OR lung* OR renal* OR hepatic* OR pulmonar* OR cardiac* OR pancrea* OR machine* OR normothermic* OR subnormothermic* OR hypothermic* OR ex-vivo* OR in-vivo* OR ex-situ* OR in-situ OR regional* OR cold* or warm*) NEAR/2 (perfus* OR reperfus* OR preserv* OR storage* OR repair* OR conservation*)) OR ((intervention*) NEAR/2 (donor* OR research* OR organ*)) OR NRP OR ANRP OR TANRP OR XVIVO OR OrganOx OR (control* NEAR/2 oxygenat* NEAR/2 rewarm*) OR NMP OR MP OR HMP OR EVMP OR ESMP OR ESHP OR SNMP OR organ-care-system* OR OCS OR D-HOPE OR COR OR OP-ECMO OR ECPR OR ECLS OR EISOR OR ISRP OR Persufflation* OR ((Cerebral* OR brain* OR systemic*) NEAR/2 (circulation* OR blood-flow)))) AND (TS=((economic* NEAR/2 evaluat*) OR ((cost OR costs) NEAR/2 (benefit* OR effectiv* OR utilit* OR compar* OR ratio OR quality-of-life OR analys*)) OR low-cost OR ((quality OR disablit* OR wellbeing OR well-being) NEAR/2 adjust* NEAR/2 (life-year* OR lifeyear*)) OR daly* OR qaly* OR waly* OR waiting-list* OR ((health OR biomedical*) NEAR/2 technolog* NEAR/2 assessment*) OR hta) OR TI=(economic* OR cost OR costs)) NOT DT=(Meeting Abstract OR Meeting Summary) AND PY=(2015-2030)9

SDC Material Methods Table S1: Inclusion and exclusion criteria used for the title, abstract and full-text screening

Include	Exclude	Category
Solid-organ transplant (Lung, Heart, Kidney, Liver, Pancreas)	Animal studies	Population
Human studies	Paediatric patients	Population
Machine perfusion of any type (NRP, NMP, HMP, etc.)	Medical devices not related to MP	Intervention
Static cold storage (comparator)	Bridging therapies without comparison to MP	Comparator
No transplant (as comparator where relevant)	Case description only (medical)	Comparator
Clinical outcomes	Clinical trials without QoL or cost outcomes	Outcome
ICER, Costs	Early phase clinical trials	Economic Outcomes
QoL/HRQoL	Commentaries, opinion pieces	Patient-Reported Outcomes
Mid- to long-term outcomes (e.g., survival beyond 7 days)	Protocols (if study has already been published)	Outcome Time Horizon
Primary data in clinical studies	Reviews	Study Design
Full-text available		Accessibility
Cost-effectiveness study		Study Type – Economic
Headroom analyses		Study Type – Economic
Other studies that include cost		Study Type – Economic

SDC Results Table S2: Costs included in economic evaluations

The following tables present the cost components included in each study, separated by their perspective. “X” indicates that the cost component was explicitly included in the analysis. “?” indicates that inclusion of the cost component was unclear based on the information reported. “–” indicates that the cost component could not be identified in the main text or supplementary materials.

SDC Results Table S2.1: Healthcare perspective costs

Perspective stated? (Y/N)		Y	Y	Y	Y
Study characteristics	Study perspective	Healthcare			
	Author	Fisher et al. 2016	McMeekin et al. 2019	Javanbakht et al. 2021	Zimmermann et al. 2022
	Organ	Lung	Lung	Liver	Liver
Waiting list cost		X	X	_	X
Transplantation cost	Donor assessment	X	X	_	_
	Cost/expenditure donor/recipient characteristics	–	–	–	–
	Retrieval	X	X	_	_
	Recipient preparation	X	X	_	_
	Perfusion procedure: Operating room, equipment, consumables, drugs staff)	X	X	X	X
	Transplant (anaesthetic room, staffing, medication, operating room)	X	X	X	X
	Cost of dying after transplantation during same admission	–	–	–	–
	Cost of transplant with complication	_	_	_	X

	Cost 2nd transplant	_	_	_	X
Transplant cost year 1	Inpatient post-operative care (ICU, ward), e.g. staffing, consultations	X	X	X	_
	Testing/Diagnostics/monitoring	X	X	_	_
	Supportive care (e.g. cardiac and respiratory support)	X	X	_	_
	Medication (immunosuppressive, concomitant)	X	X	X	_
	Consumables & Equipment	X	X	_	_
	Complications	X	X	X	X
	Unplanned hospital admission	X	X	X	_
	Out-patient care at clinic	X	X	X	_
	Out-patient care at GP	X	X	X	_
	"Cost alive with transplant"	_	_	_	X
Transplant cost year 2	Out-patient care at clinic	X	X	X	_
	Out-patient care at GP	X	X	X	_
	Complications	X	X	X	X
	Medication (e.g. Immunosuppressants)	X	X	X	_
Transplant cost year 3 onwards		X	X	X	_

SDC Results Table S2.2: Institutional perspective costs

Perspective stated? (Y/N)		N	N	N	N	Y	N	Y, discussion	Y, abstract	Y	Y	N	Y	N
Study characteristics	Study perspective	Institutional												
	Author	Raigani et al. 2020	Wehrle et al. and Gross 2024	Wehrle et al. and Hong 2024	Wehrle et al. and Zhang 2024	Wehrle, Satish et al. 2025	Halpern et al. 2021	Kent et al. 2024	Urban et al. 2023	Endo et al. 2024	Rayar et al. 2020	Risbey et al. 2025	Peel et al. 2023	Yin et al. 2025
	Organ	Liver	Liver	Liver	Liver	Liver	Lung	Lung	Heart	Liver	Liver	Liver	Lung	Lung
Waiting list cost		–	–	X	X (90-day pre Tx cost)	–	–	–	–	–	–	–	Monthly cost per phase'	–
Transplantation cost	Donor assessment	–	–	–	–	?	–	‘Transplant encounter'	Flat fee	–	–	–	–	–
	Cost/expenditure donor/recipient characteristics	–	–	–	–	–	–		–	–	–	–	Predictors of cost'	–
	Retrieval	–	X	–	X	X	X		Flat fee	–	–	–	–	–
	Recipient preparation	–	?	?	?	?	?		?	–	–	–	–	?
	Perfusion procedure: Operating room, equipment, consumables, drugs staff)	X	X	X	X	X	X		X	X	X	X	X	Total inpatient hospital cost'

	Transplant (anaesthetic room, staffing, medication, operating room)	X	X	X	X	X	X		X	X	X	–	X	Total in patient hospital cost'
	Cost of dying after transplantation during same admission	–	–	?	–	–	–		–	–	–	–	–	–
	Cost of transplant with complication	N/A	90 days global cost	X	?	–	–		–	–	X DRG	–	?	total in patient hospital cost
	Cost 2nd transplant	N/A		?	–	–	–		–	X	?	–	?	?
Transplant cost year 1	Inpatient post-operative care (ICU, ward), e.g. staffing, consultations	N/A		X Assumed to be within the cost obtained for patients in hospital	May be included in the 90-day post-transplant cost, Vlavien Dindo grade for complications included	X	X	X" 1-year aggregate incl. recipients who died prior to 1-year follow up"	X	X	X DRG	–	X	total in patient hospital cost
	Testing/Diagnostics/monitoring	N/A				May be included in the 90-180 day post-Tx	X		X	X		–	In-patient cost	
	Supportive care (e.g. cardiac and respiratory support)	N/A					X		?	–		–		
	Medication (immunosupressive, concomitant)	N/A					X		X	X (during hospital admission)		–		

	Consumables & Equipment	N/A					X		X	X		–		
	Complications	N/A					–		–	X		–		
	Unplanned hospital admission	N/A					–		–	?		–		
	Out-patient care at clinic	N/A		?			X (PT/OT/Speech)		X Rehabilitation	X		–	X	–
	Out-patient care at GP	N/A		–	N/A	N/A	–		–	–	–	–	–	–
	"Cost alive with transplant"	N/A		–	90-day	90-180 day	–		–	–	–	–	–	–
Transplant cost year 2	Out-patient care at clinic	–	–	–	–	–	–	–	–	–	–	–	X	–
	Out-patient care at GP	–	–	–	–	–	–	–	–	–	–	–	–	–
	Complications	–	–	–	–	–	–	–	–	–	–	–	?	–
	Medication (e.g. Immunosupressants)	–	–	–	–	–	–	–	–	–	–	–	?	–
Transplant cost year 3 onwards		–	–	–	–	–	–	–	–	–	–	–	X	–

SDC Results Table S2.3: Payer perspective costs

Perspective stated? (Y/N)		Y	Y	Y	Y	Y	Y, appendix	Y	Y
**Study characteristics**	Study perspective	Payer							
	Author	Axelrod et al. 2019	Tedesco Silva et al. 2018	Tedesco Silva et al. 2024	Ontario Health (Quality) 2020	Handley et al. 2023	Pradat et al. 2023	Webb et al. 2021	Webb et al. 2022
	Organ	Kidney	Kidney	Kidney	Heart	Liver	Liver	Liver	Liver
	Waiting list cost	_	_	X	X	X	_	_	_
Transplantation cost	Donor assessment	_	_	X	_	?	_	_	_
	Cost/expenditure donor/recipient characteristics	X	_	_	_	?	_	_	_
	Retrieval	_	_	X	X	?	?	X	_
	Recipient preparation	_	_	?	?	?	?	_	_
	Perfusion procedure: Operating room, equipment, consumables, drugs staff)	_	X	X	X	X	X	X	X
	Transplant (anaesthetic room, staffing, medication, operating room)	_		X	X	X	X	X	X

	Cost of dying after transplantation during same admission	–	–	–	–	–	–	–	Survive post-transplant cost per year bundled
	Cost of transplant with complication	X DGF	X DGF, return to dialysis	?	X	?	?	–	
	Cost 2nd transplant	–	–	–	–	X	?	–	
Transplant cost year 1	Inpatient post-operative care (ICU, ward), e.g. staffing, consultations	X	–	X	X	Bundled	X	–	
	Testing/Diagnostics/monitoring	–	–	X	X		DRG	–	
	Supportive care (e.g. cardiac and respiratory support)	–	–	X dialysis	X			–	
	Medication (immunosupressive, concomitant)	–	Cost of kidney transplant maintenance first year'	X	X			–	
	Consumables & Equipment	–		X	X			–	
	Complications	–		?	X			–	
	Unplanned hospital admission	–		?	?			–	
	Out-patient care at clinic	–		X	X		?	–	
	Out-patient care at GP	–	–	–	–	–	–	–	

	"Cost alive with transplant"	_	X	_	_	X	_	_	
Transplant cost year 2	Out-patient care at clinic	_	_	X	X	Bundled	_	_	
	Out-patient care at GP	–	–	–	–	–	–	–	
	Complications	_	_	?	X	Bundled	_	_	
	Medication (e.g. Immunosupressants)	_	_	X	X		_	_	
Transplant cost year 3 onwards		_	_	X	X	Bundled	_	_	

SDC Results Table S3: Discard and acceptance rates of organs, reported in included studies

Author	Summary
**Chapman et al. 2023**	NMP: 29.2% (56/192); SCS: 31.9% (61/191)
**Halpern et al. 2021**	Acceptance: 81–87% for ECD lungs with MP vs. 78% without
**Handley et al. 2023**	5.8% increase in annual organ yield with MP
**Javanbakht et al. 2020**	12.4% higher utilization with MP
**Nasralla et al. 2018**	32 livers discarded with MP vs. 16 discarded without (50% reduction)
**Nilsson et al. 2019**	Up to 50% of lungs accepted with MP vs. 27% without
**Ontario et al. 2020**	DCD heart use rate with MP: 83%
**Raigani et al. 2020**	398 additional livers transplanted using MP (viability of rejected organs: 55%)
**Zeriouha et al. 2016**	7/14 marginal organs transplanted with MP vs. 0 without
**Zimmermann et al. 2022**	85% of livers assumed utilized in both MP and comparator arms
**Tedesco Silva et al. 2024**	MP associated with 1123 additional ECD kidney transplants
**Ontario Health 2020**	17% of DCD hearts on MP declined (i.e., 83% used)

SDC Results Table S4: Characteristics: Economic studies

Type of Organ	Author and year of publication	Study perspective	Method	Model type	Time horizon	Cycle length	Health states		
							Names	Nr.	Waitlist Y/NA/N
Kidney	Axelrod et al. 2019	Provider (UHC)/ payer (Medicare) perspective (Payer)	Cost-effectiveness	_	_	_	_	N/A	N/A
	Tedesco Silva et al. 2018	SUS (Payer)	Relative cost-effectiveness	Decision tree	1 year	_	Immediate functioning, failure, DGF 1-year post-transplant, return to dialysis	4	N
	Tedesco Silva et al. 2024	Public payer	Cost effectiveness	Markov model	5 years	1-year	waitlist, death on waitlist, SCD Kidney tx, ECD kidney tx, dead or alive	6	Y
Heart	Ontario Health (Quality) 2020	Public payer	Net budget impact	Flow-chart schematic	_	_	_	N/A	N/A
	Urban et al. 2023	Institutional	Financial impact	_	5 years	_	_	N/A	N/A
Liver	Endo et al. 2024	Institutional	Cost analysis	_	_	_	_	N/A	N/A
	Handley et al. 2023	Healthcare payer perspective (Paye r)	Cost-utility	Markov model and decision tree	10 years	1 year	(1) waitlist, transplanted with an SCS graft, survival post-transplant, and death. (2) waitlist, transplanted with an SCS graft, transplanted with an NMP graft, survival posttransplant (SCS), survival post-transplant (NMP), and death	4 and 6	Y
	Javanbakht et al. 2021	Healthcare	Cost-utility	Markov model and decision tree	Lifetime	1 Year	wait-ing list, alive, alive with complications, and dead	4	Y
	Pradat et al. 2023	Public health insurance (Payer)	RCT, Cost-effectiveness analysis protocol	_	1-year	_	_	N/A	N/A
	Raigani et al. 2020	Institutional	Sum of direct and indirect costs	_	_	_	_	N/A	N/A
	Rayar et al. 2020	Institutional	Cost effectiveness	_	_	_	_	N/A	N/A

	Webb et al. 2021	Single-payer	Cost-analysis	_	_	_	_	N/A	N/A
	Webb et al. 2022	Public healthcare payer (Payer)	Cost-utility	Markov model and decision tree	5 years	1 year	Strategy 1: waitlist, transplanted with a SCS graft, survive post-transplant, and dead. Strategy 2: waitlist, transplanted with a NMP graft, transplanted with a SCS graft, survive post-transplant, and dead	4 and 5	Y
	Wehrle et al. and Gross 2024	Institutional	Descriptive analysis of cost and outcomes		90 days	_	_	N/A	N/A
	Wehrle et al. and Hong 2024	Institutional	Cost effectiveness	_	_	_	_	N/A	N/A
	Wehrle et al. and Zhang 2024	Institutional	Cost-effectiveness	_	_	_	_	N/A	N/A
	Zimmermann et al. 2022	Healthcare	Cost-utility	Markov model	Lifetime	1 month	Waitlist, Receive a transplant, dead , no complications, a biliary complication, Early Allograft Dysfunction (EAD) or Primary Non-Function (PNF).	8	Y
	Wehrle, Satish et al. 2025	Institutional	Cost-effectiveness	Decision analytic model	6 months	90 days	Clavien Dindo (CD) 1-5	Grouped, CD 1-5 (2 or 5)	N
	Risbey et al. 2025	Institutional	Cost-analysis	N/A	8 years	N/A	N/A	N/A	N/A
	Fisher et al. 2016	Healthcare	Cost-utility	Markov model	Lifetime	1 year	Waiting list, removed from the waiting list, dying , receiving a standard, EVLP lung transplant, survive 1 year post lung transplant, post-lung transplant state	6	Y
**Lung**	Halpern et al. 2021	Institutional	Cost-effectiveness	_	_	_	_	N/A	N/A
	Kent et al. 2024	Institutional	Cost effectiveness	_	1 year	_	_	N/A	N: Missing data for Markov model

McMeekin et al. 2019	Healthcare (NHS)	Cost-utility	Markov model	Lifetime	1-year	Waiting list, removed from the waiting list, Death, Receiving a standard LTx, Receiving an EVLP LTx'. Post-transplant	6 or 9	Y
Peel et al. 2023	Institutional	Retrospective cost-analysis	Multi-state model	5 years	10 days	Referral, WL, transplant hospitalization, post-tx year 1&2, 3+, dead, peri-mortem between each state to dead	10	Y
Yin et al. 2025	Institutional	Cost Effectiveness	N/A	N/A	N/A	N/A	N/A	N/A

SDC Results Table S5: Availability, reference and measurement method for health-related quality of life

Author	Study type	Organ type	Reference Utility Values	Instrument
**Tedesco Silva Jra et al. 2018**	CEA	Kidney	Estimates, Evans et al. 1985	Not accessible
**Handley et al. 2023**	CEA	Liver	Ratcliffe et al. 2003	EQ5D
**Javanbakht et al. 2020**	CUA	Liver	Ratcliffe et al. 2003	SF-36 and EQ5D
**Webb et al. 2022**	CUA	Liver	Ratcliffe et al. 2003	EQ5D
**Zimmermann et al. 2022**	CUA	Liver	Ratcliffe et al. 2003	EQ5D
**Fischer et al. 2016**	HTA	Lung	DEVELOP UK 2016 Lung	SF-36
**McMeekin et al. 2019**	Cost-analysis	Lung	DEVELOP UK 2016 Lung	SF-36
**Kent et al. 2024**	Cost-outcome analysis	Lung	Kent et al. 2024	KPS

Study types refer to the form of economic evaluation conducted. Reference utility values indicate the source used for health-related quality of life measurements. Instruments describe the tool used to derive utility values. Abbreviations: HTA = Health Technology Assessment; CEA = Cost-Effectiveness Analysis; CUA = Cost-Utility Analysis; NA = Not Available; SF-36 = Short Form Health Survey-36; EQ5D = EuroQol 5-Dimension Questionnaire; KPS = Karnofsky Performance Status

SDC Results Table S6: Clinical outcomes measured in all included studies

Type of organ	Author and year	Survival							Other outcomes and complications						
		1-month	3-month	6-month	1-year	2-year	5-year survival	LoS (ICU, Hospital)	PNF	EAD	DGF	Graft survival (12-months)	Re-transplant	Graft rejection	Graft loss
Kidney	O'Callaghan et al. 2016	_	_	_	X	_	_	_	_	_	X	_	_	_	_
	Axelrod et al. 2019	_	_	_	_	_	_	_	_	_	X	_	_	_	_
	Tedesco Silva et al. 2018	_	_	_	X	_	_	_	X		X	X	_	_	_
	Tedesco Silva et al. 2024	-	-	-	-	-	X	-	-	-	X	-	-	-	-
Heart	Ontario HTA 2020	_	_	_	X	_	X	XX	_	_	_	_	_	X	_
	Urban et al. 2023	_	X	_	_	_	_	XX	_	_	_	_	_	_	_
Liver	Abbas et al 2024	X	X	X	X			-, X				X			
	Czigany et al. 2021	_	_	_	X	_	_	XX	_	X		X	X	X	X
	De Vries et al. 2019	7-day	X	X	X	_	_	_	X	X	_	X	_	_	_
	Hann et al. 2022	_	X	X	_	_	_	X,-	X	X	_	_	_	_	_
	Liu et al. 2021	_	_	_	_	_	_	_	_	X	_	_	_	_	_
	Markmann et al. 2022	X	_	_	_	_	_	_	_	X	_	_	_	_	_
	Nasralla et al. 2018	X	_	X	X		_	X,X	X	X	_	X	X	_	X
	Taylor et al. 2018	_	X	_	X	_	X	_	X	_	_	X	X	_	X
	Toro-Díaz et al. 2015	_	_	_	_	_	_	_	_	_	_	_	_	_	_
	Chapman et al. 2023	X	X	X	X	_	_	XX	_	X	_	X	_	_	_

	Handley et al. 2023	_	_	_	X	_	_	X,-	_	_	_	_	X	_	_
	Javanbakht et al. 2021	_	_	_	X	X	X	XX	_	X	_	_	_	_	_
	Li et al. 2024	_	_	_	_	_	_	_	_	_	_	_	_	_	_
	Pradat et al. 2023	7-day	X	_	X	_	_	X	_	X	_	_	_	_	X
	Raigani et al. 2020	_	_	_	_	_	_	_	_	_	_	_	_	_	_
	Rayar et al. 2020	_	X	_	X	_	_	XX	X	X	_	X	X	_	X
	Webb et al. 2021	_	_	_	_	_	_	_	_	_	_	_	_	_	_
	Webb et al. 2022	_	_	_	X	_	X	_	_	_	_	_	_	_	_
	Wehrle et al. 2024 Gross	_	X	_	_	_	_	XX	_	_	_	_	_	_	X
	Wehrle et al. 2024 Hong	_	_	X	X	_	_	_	X	_	_	_	_	_	X
	Wehrle et al. 2024 Zhang	_	_	X	_	_	_	_	_	_	_	_	_	_	X
	Zimmermann et al. 2022	_	_	X	_	_	_	X,-	X	X	_	_	X	_	X
	Endo et al. 2024	_	_	_	X	_	_	_	_	_	_	_	_	_	_
	Wehrle, Satish et al. 2025	_	_	X	_	_	_	-,X	X*	_	_	_	_	_	X
	Risbey et al. 2025	_	_	X	_	_	_	XX	X*	X	_	_	_	_	X
**Lung**	Fildes et al. 2015	X	X	_	X	_	_	XX	_	_	_	_	_	X	_
	Nilsson et al. 2019	_	_	_	X		_	X, -	_		_	_		_	X
	Peel et al. 2023	_	_	_	_		_	X,-	X		_	_		_	_
	Van Raemdonck et al. 2019	X	_	_	X	_	X	_	_	_	_	_	_	X	_

	Zeriouha et al. 2016	X	_	X	X		_	XX	X		_	_	X	X	_
	Fisher et al. 2016	X	_	_	X	_	_	XX	X	_	_	_	_	X	_
	McMeekin et al. 2019	X	X	_	X	_	_	X,-	X	_	_	X	_	_	_
	Peel et al. 2023 (Keshavjee)	_	_	_	_		X	X,-	_		_	_		_	_
	Halpern et al. 2021	X	X	_	_	_	_	X,-	_	_	_	_	_	X	_
	Kent et al. 2024	_	_	_	X	_	_	XX	_	_	_	_	_	_	_
	Yin et al. 2025	_	_	_	_	_	_	-,X	_	_	_	_	_	X	_
**Pancreas**	Richards et al. 2021	_	_	_	X	X	X	_	_	_	X	X	_	_	_

*nonanatomic (NAS and anastomotic strictures (AS) included

SDC: References (included studies)

S1. O'Callaghan JM, Knight SR, Morgan RD, Morris PJ. A national registry analysis of kidney allografts preserved with marshall's solution in the united kingdom. Transplantation. 2016;100(11):2447–2452. https://pubmed.ncbi.nlm.nih.gov/26760566/. Accessed Apr 30, 2025. doi: 10.1097/TP.0000000000001033.

S2. Taylor R, Allen E, Richards JA, et al. Survival advantage for patients accepting the offer of a circulatory death liver transplant. J Hepatol. 2019;70(5):855–865. https://pubmed.ncbi.nlm.nih.gov/30639505/. Accessed Apr 30, 2025. doi: 10.1016/j.jhep.2018.12.033.

S3. Liu Q, Del Prete L, Hassan A, et al. Two pumps or one pump? A comparison of human liver normothermic machine perfusion devices for transplantation. Artif Organs. 2022;46(5):859–866. https://pubmed.ncbi.nlm.nih.gov/34904245/. Accessed Apr 30, 2025. doi: 10.1111/aor.14150.

S4. Hann A, Lembach H, Nutu A, et al. Outcomes of normothermic machine perfusion of liver grafts in repeat liver transplantation (NAPLES initiative). Br J Surg. 2022;109(4):372–380. https://pubmed.ncbi.nlm.nih.gov/35170730/. Accessed Apr 30, 2025. doi: 10.1093/bjs/znab475.

S5. Abbas SH, Ceresa CDL, Hodson L, et al. Defatting of donor transplant livers during normothermic perfusion—a randomised clinical trial: Study protocol for the DeFat study. Trials. 2024;25(1):386. https://doi.org/10.1186/s13063-024-08189-4. Accessed Apr 30, 2025. doi: 10.1186/s13063-024-08189-4.

S6. de Vries Y, Berendsen TA, Fujiyoshi M, et al. Transplantation of high-risk donor livers after resuscitation and viability assessment using a combined protocol of oxygenated hypothermic, rewarming and normothermic machine perfusion: Study protocol for a prospective, single-arm study (DHOPE-COR-NMP trial). BMJ Open. 2019;9(8):e028596. https://pubmed.ncbi.nlm.nih.gov/31420387/. Accessed Apr 30, 2025. doi: 10.1136/bmjopen-2018-028596.

S7. Nasralla D, Coussios CC, Mergental H, et al. A randomized trial of normothermic preservation in liver transplantation. Nature. 2018;557(7703):50–56. https://pubmed.ncbi.nlm.nih.gov/29670285/. Accessed Apr 30, 2025. doi: 10.1038/s41586-018-0047-9.

S8. Czigany Z, Pratschke J, Froněk J, et al. Hypothermic oxygenated machine perfusion reduces early allograft injury and improves post-transplant outcomes in extended criteria donation liver transplantation from donation after brain death: Results from a multicenter randomized controlled trial (HOPE ECD-DBD). Ann Surg. 2021;274(5):705–712. https://pubmed.ncbi.nlm.nih.gov/34334635/. Accessed Apr 30, 2025. doi: 10.1097/SLA.0000000000005110.

S9. Markmann JF, Abouljoud MS, Ghobrial RM, et al. Impact of portable normothermic blood-based machine perfusion on outcomes of liver transplant: The OCS liver PROTECT randomized clinical trial. JAMA Surgery. 2022;157(3):189–198. https://doi.org/10.1001/jamasurg.2021.6781. Accessed Apr 30, 2025. doi: 10.1001/jamasurg.2021.6781.

S10. Chapman WC, Barbas AS, D'Alessandro AM, et al. Normothermic machine perfusion of donor livers for transplantation in the United States: A randomized controlled trial. Ann Surg. 2023;278(5):e912–e921. https://pubmed.ncbi.nlm.nih.gov/37389552/. Accessed Apr 30, 2025. doi: 10.1097/SLA.0000000000005934.

S11. Fildes JE, Archer LD, Blaikley J, et al. Clinical outcome of patients transplanted with marginal donor lungs via ex vivo lung perfusion compared to standard lung transplantation. Transplantation. 2015;99(5):1078–1083. https://pubmed.ncbi.nlm.nih.gov/25757211/. Accessed Apr 30, 2025. doi: 10.1097/TP.0000000000000462.

S12. Zeriouh M, Sabashnikov A, Mohite PN, et al. Utilization of the organ care system for bilateral lung transplantation: Preliminary results of a comparative study. Interact Cardiovasc Thorac Surg. 2016;23(3):351–357. https://pubmed.ncbi.nlm.nih.gov/27221998/. Accessed Apr 30, 2025. doi: 10.1093/icvts/ivw135.

S13. Van Raemdonck D, Keshavjee S, Levvey B, et al. Donation after circulatory death in lung transplantation—five-year follow-up from ISHLT registry. J Heart Lung Transplant. 2019;38(12):1235–1245. https://pubmed.ncbi.nlm.nih.gov/31777330/. Accessed Apr 30, 2025. doi: 10.1016/j.healun.2019.09.007.

S14. Nilsson T, Wallinder A, Henriksen I, et al. Lung transplantation after ex vivo lung perfusion in two Scandinavian centres. Eur J Cardiothorac Surg. 2019;55(4):766–772. https://www.ncbi.nlm.nih.gov/pmc/articles/PMC6421510/. doi: 10.1093/ejcts/ezy354.

S15. Peel JK, Pullenayegum EM, Naimark D, et al. Evaluating the impact of ex vivo lung perfusion on organ transplantation. Ann Surg. 2023;278(2):288–296. https://www.ncbi.nlm.nih.gov/pmc/articles/PMC10321509/. doi: 10.1097/SLA.0000000000005887.

S16. Richards JA, Roberts JL, Fedotovs A, et al. Outcomes for circulatory death and brainstem death pancreas transplantation with or without use of normothermic regional perfusion. Br J Surg. 2021;108(12):1406–1408. https://pubmed.ncbi.nlm.nih.gov/34155506/. Accessed Apr 30, 2025. doi: 10.1093/bjs/znab212.

S17. Tedesco Silva H, Evans RW, Gavaghan MB, Vazquez VC. A cost-effectiveness analysis of organ preservation methods for deceased donor kidneys at high risk for delayed graft function in Brazil. Transplant Proc. 2018;50(10):3121–3127. https://pubmed.ncbi.nlm.nih.gov/30577177/. Accessed Apr 30, 2025. doi: 10.1016/j.transproceed.2018.06.024.

S18. Axelrod DA, Malinoski D, Patel MS, et al. Modeling the economic benefit of targeted mild hypothermia in deceased donor kidney transplantation. Clin Transplant. 2019;33(7):e13626. https://pubmed.ncbi.nlm.nih.gov/31162858/. Accessed Apr 30, 2025. doi: 10.1111/ctr.13626.

S19. Tedesco Silva H, Ramos TRdM, de Carvalho DDBM, et al. Use of machine perfusion to increase the number of expanded criteria deceased donor kidney transplants: A pharmacoeconomic analysis. Transplant Direct. 2024;10(8):e1668. https://pubmed.ncbi.nlm.nih.gov/38988688/. Accessed Apr 30, 2025. doi: 10.1097/TXD.0000000000001668.

S20. Ontario Health (Quality). Portable normothermic cardiac perfusion system in donation after cardiocirculatory death: A health technology assessment. Ont Health Technol Assess Ser. 2020;20(3):1–90. https://pubmed.ncbi.nlm.nih.gov/32190164/. Accessed Apr 30, 2025.

S21. Urban M, Ryan TR, Um JY, Siddique A, Castleberry AW, Lowes BD. Financial impact of donation after circulatory death heart transplantation: A single-center analysis. Clin Transplant. 2024;38(4):e15296. https://pubmed.ncbi.nlm.nih.gov/38545928/. Accessed Apr 30, 2025. doi: 10.1111/ctr.15296.

S22. Raigani S, De Vries RJ, Carroll C, et al. Viability testing of discarded livers with normothermic machine perfusion: Alleviating the organ shortage outweighs the cost. Clin Transplant. 2020;34(11):e14069. https://pubmed.ncbi.nlm.nih.gov/32860634/. Accessed Apr 30, 2025. doi: 10.1111/ctr.14069.

S23. Rayar M, Beaurepaire J, Bajeux E, et al. Hypothermic oxygenated perfusion improves extended criteria donor liver graft function and reduces duration of hospitalization without extra cost: The PERPHO study. Liver Transplant. 2021;27(3):349.

https://journals.lww.com/lt/fulltext/2021/03000/hypothermic_oxygenated_perfusion_improves_extended.9.aspx. Accessed Apr 30, 2025. doi: 10.1002/lt.25955.

S24. Javanbakht M, Mashayekhi A, Trevor M, Branagan-Harris M, Atkinson J. Cost-utility analysis of normothermic liver perfusion with the OrganOx metra compared to static cold storage in the United Kingdom. J Med Econ. 2020;23(11):1284–1292. https://pubmed.ncbi.nlm.nih.gov/32729749/. Accessed Apr 30, 2025. doi: 10.1080/13696998.2020.1804391.

S25. Webb AN, Izquierdo DL, Eurich DT, Shapiro AMJ, Bigam DL. The actual operative costs of liver transplantation and normothermic machine perfusion in a Canadian setting. Pharmacoecon Open. 2021;5(2):311–318. https://pubmed.ncbi.nlm.nih.gov/33190212/. Accessed Apr 30, 2025. doi: 10.1007/s41669-020-00241-8.

S26. Webb AN, Lester ELW, Shapiro AMJ, Eurich DT, Bigam DL. Cost-utility analysis of normothermic machine perfusion compared to static cold storage in liver transplantation in the Canadian setting. Am J Transplant. 2022;22(2):541–551. https://pubmed.ncbi.nlm.nih.gov/34379887/. Accessed Apr 30, 2025. doi: 10.1111/ajt.16797.

S27. Zimmermann J, Carter AW. Cost-utility analysis of normothermic and hypothermic ex-situ machine perfusion in liver transplantation. Br J Surg. 2022;109(2):e31–e32. https://pubmed.ncbi.nlm.nih.gov/34904161/. Accessed Apr 30, 2025. doi: 10.1093/bjs/znab431.

S28. Handley TJ, Arnow KD, Melcher ML. Despite increasing costs, perfusion machines expand the donor pool of livers and could save lives. J Surg Res. 2023;283:42–51. https://pubmed.ncbi.nlm.nih.gov/36368274/. Accessed Apr 30, 2025. doi: 10.1016/j.jss.2022.10.002.

S29. Endo C, van Rijn R, Huurman V, et al. Cost-effectiveness of dual hypothermic oxygenated machine perfusion versus static cold storage in DCD liver transplantation. Transplantation. 2025;109(2):e101–e108. https://pubmed.ncbi.nlm.nih.gov/39853733/. Accessed Apr 30, 2025. doi: 10.1097/TP.0000000000005232.

S30. (a) Wehle CJ, Gross A, Fares S, et al. Changing landscape of open offers in liver transplantation in the machine perfusion era: Exposure, equity, and economics. Clin Transplant. 2024;38(10):e70012. https://pubmed.ncbi.nlm.nih.gov/39460610/. Accessed Apr 30, 2025. doi: 10.1111/ctr.70012.

S31. (b) Wehrle CJ, Hong H, Gross A, et al. The impact of normothermic machine perfusion and acuity circles on waitlist time, mortality, and cost in liver transplantation: A multicenter experience. Liver Transpl. 2025;31(4):438–449. https://pubmed.ncbi.nlm.nih.gov/38833290/. Accessed Apr 30, 2025. doi: 10.1097/LVT.0000000000000412.

S32. (c) Wehrle CJ, Zhang M, Khalil M, et al. Impact of back-to-base normothermic machine perfusion on complications and costs: A multicenter, real-world risk-matched analysis. Ann Surg. 2024;280(2):300–310. https://pubmed.ncbi.nlm.nih.gov/38557793/. Accessed Apr 30, 2025. doi: 10.1097/SLA.0000000000006291.

S33. Wehrle CJ, Satish S, Dewey E, et al. A new era of decision-making in liver transplantation: A prospective validation and cost-effectiveness analysis of FMN-guided liver viability assessment during normothermic machine perfusion. *Annals of Surgery*. 2025;282(3):479.https://journals.lww.com/annalsofsurgery/fulltext/2025/09000/a_new_era_of_decision_making_in_liver.14.aspx. Accessed Sep 4, 2025. doi: 10.1097/SLA.0000000000006822.

S34. Pradat P, Pantel S, Maynard M, et al. End-ischemic hypothermic oxygenated perfusion for extended criteria donors in liver transplantation: A multicenter, randomized controlled trial—HOPExt. Trials. 2023;24(1):379. https://doi.org/10.1186/s13063-023-07402-0. Accessed Apr 30, 2025. doi: 10.1186/s13063-023-07402-0.

S35. Risbey CWG, Babekuhl D, Yousif P, et al. A novel, institutionally developed hypothermic oxygenated machine perfusion system allows low-cost, universal implementation for liver transplantation: A safety and feasibility pilot study. Liver Transpl. 2025; :10.1097/LVT.0000000000000686.https://journals.lww.com/lt/abstract/9900/a_novel,_institutionally_developed_hypothermic.676.aspx. Accessed Sep 4, 2025. doi: 10.1097/LVT.0000000000000686.

S36. Fisher A, Andreasson A, Chrysos A, et al. An observational study of donor ex vivo lung perfusion in UK lung transplantation: DEVELOP-UK. Health Technol Assess. 2016;20(85):1–276. https://pubmed.ncbi.nlm.nih.gov/27897967/. Accessed Apr 30, 2025. doi: 10.3310/hta20850.

S37. McMeekin N, Chrysos AE, Vale L, Fisher AJ. Incorporating ex-vivo lung perfusion into the UK adult lung transplant service: An economic evaluation and decision analytic model. BMC Health Serv Res. 2019;19(1):326. https://doi.org/10.1186/s12913-019-4154-6. Accessed Apr 30, 2025. doi: 10.1186/s12913-019-4154-6.

S38. Halpern SE, Kesseli SJ, Au S, et al. Lung transplantation after ex vivo lung perfusion versus static cold storage: An institutional cost analysis. Am J Transplant. 2021;22(2):552–564. https://pubmed.ncbi.nlm.nih.gov/34379885/. Accessed Apr 30, 2025. doi: 10.1111/ajt.16794.

S39. Peel JK, Pullenayegum EM, Naimark D, et al. Evaluating the impact of ex vivo lung perfusion on organ transplantation. Ann Surg. 2023;278(2):288–296. https://www.ncbi.nlm.nih.gov/pmc/articles/PMC10321509/. Accessed Apr 30, 2025. doi: 10.1097/SLA.00000000000040.

S40. Kent J, Nordgren R, Ahn D, et al. Cost effectiveness of commercial portable ex vivo lung perfusion at a low-volume US lung transplant center. Artif Organs. 2024;48(11):1288–1296. https://pubmed.ncbi.nlm.nih.gov/38924545/. Accessed Apr 30, 2025. doi: 10.1111/aor.14816.

S41. Yin V, Atay SM, Rodman JCS, et al. Impact of ex vivo lung perfusion on inpatient cost: A propensity score-matched analysis of the US nationwide healthcare cost and utilization project database. Clin Transplant. 2025;39(2):e70096. https://pubmed.ncbi.nlm.nih.gov/39876610/. Accessed Sep 4, 2025. doi: 10.1111/ctr.70096.

42. Toro-Díaz H, Mayorga ME, Barritt AS, Orman ES, Wheeler SB. Predicting liver transplant capacity using discrete event simulation. *Med Decis Making*. 2015;35(6):784–796.https://pubmed.ncbi.nlm.nih.gov/25391681/. Accessed Apr 30, 2025. doi: 10.1177/0272989X14559055.

43. Li Z, Pfister M, Huwyler F, et al. Revolutionizing liver transplantation: Transitioning to an elective procedure through ex situ normothermic machine perfusion - A benefit analysis. *Ann Surg*. 2024;280(5):887–895.https://pubmed.ncbi.nlm.nih.gov/39077782/. Accessed Apr 30, 2025. doi: 10.1097/SLA.0000000000006462.
